# Curcumin and Its Potential to Target the Glycolytic Behavior of Lactate-Acclimated Prostate Carcinoma Cells with Docetaxel

**DOI:** 10.3390/nu16244338

**Published:** 2024-12-16

**Authors:** Dongsic Choi, Jun Gi Lee, Su-Hak Heo, Moon-Kyen Cho, Hae-Seon Nam, Sang-Han Lee, Yoon-Jin Lee

**Affiliations:** 1 Department of Biochemistry, College of Medicine, Soonchunhyang University, Cheonan 31511, Republic of Korea; dongsic@sch.ac.kr (D.C.); m1037624@sch.ac.kr (S.-H.L.); 2 Biochemistry and Molecular Biology, Marquette University, Milwaukee, WI 53233, USA; jungi0714@gmail.com; 3 Department of Medicinal Bioscience, College of Biomedical and Health Science, Konkuk University Glocal Campus, Chungju 27478, Republic of Korea; cableshh@gmail.com; 4 Division of Molecular Cancer Research, Soonchunhyang Medical Research Institute, Soonchunhyang University, Cheonan 31151, Republic of Korea; mkcho@schmc.ac.kr (M.-K.C.); namhs@sch.ac.kr (H.-S.N.)

**Keywords:** curcumin, glycolysis, lactic acid, prostate cancer cells, chemoresistance, apoptosis, necroptosis

## Abstract

**Background:** Dysregulated cellular metabolism is known to be associated with drug resistance in cancer treatment. **Methods:** In this study, we investigated the impact of cellular adaptation to lactic acidosis on intracellular energy metabolism and sensitivity to docetaxel in prostate carcinoma (PC) cells. The effects of curcumin and the role of hexokinase 2 (HK2) in this process were also examined. **Results:** PC-3AcT and DU145AcT cells that preadapted to lactic acid displayed increased growth behavior, increased dependence on glycolysis, and reduced sensitivity to docetaxel compared to parental PC-3 and DU145 cells. Molecular analyses revealed activation of the c-Raf/MEK/ERK pathway, upregulation of cyclin D1, cyclin B1, and p-cdc2Thr161, and increased levels and activities of key regulatory enzymes in glycolysis, including HK2, in lactate-acclimated cells. HK2 knockdown resulted in decreased cell growth and glycolytic activity, decreased levels of complexes I–V in the mitochondrial electron transport chain, loss of mitochondrial membrane potential, and depletion of intracellular ATP, ultimately leading to cell death. In a xenograft animal model, curcumin combined with docetaxel reduced tumor size and weight, induced downregulation of glycolytic enzymes, and stimulated the upregulation of apoptotic and necroptotic proteins. This was consistent with the in vitro results from 2D monolayer and 3D spheroid cultures, suggesting that the efficacy of curcumin is not affected by docetaxel. **Conclusions:** Overall, our findings suggest that metabolic plasticity through enhanced glycolysis observed in lactate-acclimated PC cells may be one of the underlying causes of docetaxel resistance, and targeting glycolysis by curcumin may provide potential for drug development that could improve treatment outcomes in PC patients.

## 1. Introduction

Unlike normal cells, cancer cells tend to rely on the low energy yield of glycolysis to make ATP, even when sufficient levels of oxygen are present [1]. Glycolysis produces 18 times less ATP than mitochondrial oxidation, but is 100 times faster than oxidative phosphorylation, which may benefit cancer cells by providing them with greater energy [2]. Additionally, the provision of metabolic intermediates or precursors and NADPH generated from glycolysis and hexose monophosphate shunts can promote rapid cell proliferation of cancer cells and help maintain intracellular redox status, respectively [3].

High dependence on glycolysis can promote drug resistance, angiogenesis, and metastatic behavior of cancer cells by releasing excess generated protons into the extracellular space and maintaining an alkaline intracellular pH and acidic extracellular pH [4]. Lactic acid is produced intracellularly as a result of high glycolysis. It is one of the major metabolites that accumulates in the tumor microenvironment and causes acidification. Although excess lactic acid can also be utilized for energy provision, increased lactate production and subsequent acidification of the tumor microenvironment (TME) appear to promote several important malignant progressions, including angiogenesis, tissue invasion/metastasis, and drug resistance [4]. Previous studies have demonstrated that lactic acid levels in prostate cancer (PC) are closely related to cancer progression [5]. While healthy prostate cells produce ATP primarily through glucose oxidation even under aerobic conditions, prostate cells undergoing neoplastic transformation or early-stage PC use citric acid from the tricarboxylic acid cycle (TCA) cycle for oxidative phosphorylation. At advanced stages, PC cells exhibit the glycolysis-dependent Warburg phenotype, thereby increasing lactate production [6,7]. A systemic increase in lactate is associated with disease recurrence and is known to have a poor prognosis for survival [8,9]. Therefore, targeting the glycolytic pathway as a therapeutic strategy to combat cancer may provide an effective approach for the development of new targeted anticancer drugs in cancers that exhibit a prevalence of the glycolytic phenotype.

Docetaxel, a semisynthetic analog of the natural product paclitaxel, is the most widely used chemotherapeutic drug for the treatment of metastatic castrate-resistant PC (mCRPC). It preferentially binds to β-tubulin, altering cellular microtubule dynamics, thus leading to cell cycle arrest and apoptosis [10]. Although it has transient efficacy, patients receiving long-term treatment with docetaxel are known to exhibit drug resistance and systemic cytotoxicity, limiting the clinical use of taxane-based chemotherapy in CRPC [11]. PC cells are known to acquire resistance to docetaxel through tubulin alterations, enhanced survival signaling pathways, decreased drug influx and increased drug efflux, altered androgen receptor signaling, centrosome clustering, and cancer stem cells [11]. Recently, there has been increasing evidence that metabolic reprogramming is involved in docetaxel resistance in PC cells [12,13]. The Warburg effect is a type of metabolic reprogramming characterized by increased glycolysis and consequently high lactate production. This change not only allows cancer cells to adapt to various conditions of the TME by providing them with sufficient energy and metabolic intermediates essential for rapid proliferation and malignant progression, but also induces immunosuppression and immune evasion of cancer cells, making them more prone to developing resistance to chemotherapy [14,15]. Moreover, TME components plays a central role in shaping and maintaining these metabolic changes in cancer cells [16]. However, although direct evidence for metabolic reorganization and anticancer drug resistance through crosstalk between the TME and cancer cells is limited and further studies are needed, targeting altered metabolism in conjunction with chemotherapy might be a rational therapeutic strategy to improve therapeutic response and overcome drug resistance. Therefore, recent studies have attempted to develop natural-based agents that can enhance the therapeutic efficacy of conventional chemotherapies to improve the current cure rates for PCs while minimizing adverse effects on healthy cells [17].

Curcumin, a flavonoid found in the roots of turmeric or *Curcuma longa*, has been shown to have antioxidant, anti-inflammatory, and anti-proliferative properties [18]. Anti-carcinogenic effects of curcumin have been demonstrated in several cancers, including PC, by modulating various signaling pathways, including p53, mitogen-activated protein (MAP) kinase, phosphoinositol-3 kinases/protein kinase B (P13K/Akt), Janus kinase/signal transducer and activator of transcription (JAK/STAT), sonic hedgehog, and nuclear factor kappa-light-chain-enhancer of activated B cells (NF-κB) pathways [19]. Curcumin is a natural product that is less toxic and has fewer side effects than existing chemotherapy drugs and can be used safely. It is also known to be advantageous in sensitizing cancer cells when used in combination with chemotherapeutic drugs [20]. In previous studies, we first reported that the preferential cytotoxic effect of curcumin on PC-3AcT cells pre-adapted to a lactate-containing medium with increased tolerance to docetaxel was associated with its anti-glycolytic role through inhibition of MEK/ERK signaling, suggesting that there is a relationship between the dependence of PC cells on glycolysis and the effect of protecting them from chemotherapeutic drugs [21,22].

In this study, we performed the following experiments to further confirm the anticancer properties of curcumin, which showed a better killing effect on lactate-acclimated cells with an increased glycolytic and docetaxel-resistant phenotype. First, we investigated whether there were differences in cell growth, glycolysis, and bioenergetics at the basal level between lactate-acclimated PC-3AcT or DU145AcT cells and their parental PC-3 or DU145 cells. Next, by examining the knockdown effect of hexokinase 2 (HK2), a key target of curcumin’s anti-glycolytic role, we sought to elucidate the importance of this enzyme as an attractive target for the development of PC therapeutics. Finally, we evaluated whether the antiglycolytic effects of curcumin demonstrated in 2D monolayer cultures were not affected by co-treatment with docetaxel in 3D spheroid cultures and a nude mouse xenograft model.

## 2. Materials and Methods

### 2.1. Cell Culture and Assays

Human prostate epithelial cell lines HPrEC and RWPE-1 and human PC cell lines PC-3 and DU145 were purchased from the American Type Culture Collection (ATCC; Manassas, VA, USA). Acidic pre-adapted cells designated as PC-3AcT and DU145AcT were established by continuously exposing PC-3 and DU145 cells, respectively, to lactic acid (final concentration: 3.8 µM) over four passages for 15 days. Cells were seeded at a density of 10^4^ cells/well in 96-well cell culture plates and cultured in in Dulbecco’s Modified Eagles Medium (DMEM, Welgene Inc., Gyeongsan, Republic of Korea) containing lactic acid (final concentration: 3.8 µM) and 5% fetal bovine serum and cultured at 37 °C in a 5% CO_2_ incubator. They were then treated with fixed (40 μM) or increasing concentrations (0, 5, 10, 20, 40, 80, and 100 µM) of curcumin with or without docetaxel (40 nM) for the cell viability assay. Negative control cells were treated with 0.1% dimethyl sulfoxide. Cell viability was determined by MTT assay, as previously described [23]. The effect of combined treatment of the two compounds was assessed using the combination index (CI), as previously described [23]. To measure the activities of HK and pyruvate dehydrogenase (PDH), an HK Colorimetric Assay Kit (cat. no. K789-100) and PDH Activity Colorimetric Assay Kit (cat. no. K679-100) were used, respectively, according to the manufacturer’s instructions (BioVision, Inc., Milpotas, CA, USA). Glucose consumption was determined by assessing glucose contents in the culture media using a Glucose Colorimetric Assay Kit (cat. no. K606-100; Biovision, Inc., Milpotas, CA, USA) according to the manufacturer’s instructions. Intracellular ATP content was determined by measuring luminescence using a CellTiter-Glo Luminescent Cell Viability Assay Kit (Promega Corporation, Madison, WI, USA) according to the protocols provided by the manufacturer. Absorbance and luminescence values were measured with a GloMax-Multi microplate multimode reader (Promega Corporation, Madison, WI, USA).

### 2.2. Western Blotting

Cells were seeded at a density of 10^5^ cells/well in 6-well cell culture plates, and cultured in DMEM containing lactic acid (final concentration: 3.8 µM) and 5% fetal bovine serum at 37 °C in a 5% CO_2_ incubator. After combined treatment with curcumin (40 μM) and docetaxel (40 nM) for 48 h, whole cell lysates were extracted from samples obtained from cell culture and animal experiments using 1× RIPA buffer (1× PBS, 0.5% sodium deoxycholate, 1% NP-40, 0.1% sodium dodecyl sulfate, 10 mg/mL phenylmethylsulfonyl fluoride). Mitochondrial and cytosolic extracts were prepared with a Mitochondria Isolation Kit for Mammalian Cells according to the instruction provided (cat no. 89874; Thermo Scientific, Rockford, IL, USA). Protein concentration was determined with a BCA Protein Assay (cat no. 23225; Thermo Scientific). Forty micrograms of proteins were loaded onto 4–12% NuPAGE gels (Invitrogen, Carlsbad, CA, USA), separated, and transferred to a polyvinylidenefluoride membrane (Cytiva Life Sciences, Marlborough, MA, USA). Blots were blocked with 1× casein solution (cat. no; 37528; Thermo Fisher Scientific, Inc., Waltham, MA, USA) for 2 h at room temperature and then incubated with the primary antibody overnight at 4 °C. After washing three times with 1x PBS-Tween 20, horseradish peroxidase (HRP)-conjugated secondary antibodies was applied for 2 h at room temperature. The signal was visualized using an Enhanced Chemiluminescence (ECL) Detection Kit (cat. no. W1001; Promega). Densitometry was performed on blots using TINA 2.09 software (Raytest Isotopen Messgeraete GmbH, Straubenhardt, Germany) and normalized to β-actin. Oxphos human WB antibody cocktail (cat. no. 45-8199) and antibodies to cyclin B1 (cat. no. 4138), phosphorylated (p)-cyclin dependent kinase (CDK) 2, p-Cdc2^Thr161^ (cat. no. 9114) and p-cdc2^Tyr15^ (cat. no. 4539), p-mitogen-activated protein kinase 1/2 (p-MEK1/2; cat. no. 9154), p-extracellular-signal-regulated kinase 1/2 (p-ERK1/2; cat. no. 4370), ERK (cat. no. 9102), HK2 (cat. no. 2867), phosphofructokinase platelet (PFKP; cat. no. 93654), PDH (cat. no. 3205), voltage-dependent anion channel (VDAC; cat. no. 4661), α-tubulin (cat. no. 2144), p-mixed lineage kinase domain-like (p-MLKL; cat. no. 91689), MLKL (cat. no. 14993), p-receptor-interacting protein 3 (p-RIP3; cat. no. 93654), RIP3 (cat. no. 13526), B-cell lymphoma 2 (Bcl-2; cat. no. 2870), Bcl-2 associated X (Bax; cat. no. 5023), poly (ADP-ribose) polymerase (PARP; cat. no. 9542), cleaved PARP (cat. no. 9541), caspase-3 (cat. no. 14220), and cleaved caspase-3 (cat. no. 9664) were purchased from Cell Signaling Technology, Inc. (Danvers, CO, USA) and diluted 1:500. HRP-coupled goat anti-rabbit IgG (1:5000; cat. no. sc-2004), goat anti-mouse IgG (1:5000; cat. no. sc-2005), and antibodies to cyclin D1 (1:500; cat. no. SC-718), p53 (1:500; cat. no. sc-126), and MEK (1:500; cat. no. SC-436) were purchased from Santa-Cruz Biotechnology, Inc. (Dallas, TX, USA). Membranes were re-probed with antibodies to β-actin (1000; cat. no. A2228; Sigma-Aldrich, St. Louis, MO, USA), MEK1/2, ERK1/2, VDAC, α-tubulin, MLKL, and RIP3 as loading controls.

### 2.3. Mitochondrial Fractionation

Mitochondrial-enriched fractions were prepared according to the instruction of the Mitochondria Isolation Kit for Cultured Cells (cat no. 89874; Thermo Scientific). Briefly, cells (2 × 10^7^) were incubated in 800 µL of Mitochondria Isolation Reagent A on ice for 2 min, 10 µL of Mitochondria Isolation Reagent B on ice for 5 min, and 800 µL of Mitochondria Isolation Reagent C at 4 °C for 10 min. After centrifugation at 700× *g* for 10 min at 4 °C, the supernatant was transferred to a new 2.0 mL tube and centrifuged at 12,000× *g* for 15 min at 4 °C. The supernatant was stored as the cytosolic fraction. The pellet was resuspended in 500 µL Mitochondria Isolation Reagent C, and the mitochondrial pellet was obtained by centrifugation at 12,000× *g* for 5 min. This was defined as the mitochondrial fraction and stored at −80 °C for Western blot analysis.

### 2.4. Mitochondrial Membrane Potential (ΔΨ)

In each well of a 6-well culture plate, cells (1 × 10^5^) were seeded and incubated overnight in lactic acid-containing DMEM. Cells were then treated with or without 40 nM docetaxel for 48 h. After trypsinization, cells were harvested by centrifugation at 500× *g* for 7 min, adjusted to 10^6^ cells/mL, and incubated in serum-free DMEM containing 30 nM rhodamine 123 (Sigma-Aldrich) at 37 °C for 30 min in the dark. Then, the cells were centrifuged at 500× *g* for 5 min to discard the supernatant, and washed twice in pre-warmed medium. The fluorescence intensity of the cells was measured using a MACSQuant analyzer and MACSQuantify software version 2.5 (Miltenyi Biotec GmbH, Bergisch Gladbach, Germany).

### 2.5. Reactive Oxygen Species

Cells (1 × 10^5^) were seeded per well of a 6-well culture plate and incubated overnight in lactic acid-containing DMEM. Cells were then treated with or without 40 nM docetaxel for 48 h. Trypsinized cells were collected by centrifugation at 500× *g* for 7 min, adjusted to 10^6^ cells/mL, and incubated in serum-free DMEM containing 10 µM 2′,7′-dichlorodihydrofluorescein diacetate (Sigma-Aldrich) at 37 °C for 30 min in the dark, and then the reactive oxygen species (ROS) were measured. The fluorescence intensity of the cells was measured using a MACSQuant analyzer and MACSQuantify software version 2.5 (Miltenyi Biotec GmbH, Bergisch Gladbach, Germany).

### 2.6. Annexin V-PE/7-AAD Double Staining

To analyze the distribution of apoptotic and necrotic cells, the Muse Annexin V & Dead Cell Assay Kit (cat. no. MCH100105; Merck KGaA, Darmstadt, Germany) was used according to the manufacturer’s instructions. Cells were treated with or without 40 nM docetaxel for 48 h. Cells were then harvested by trypsinization and resuspended in 200 µL of Muse Annexin V & Dead Cell reagent for 30 min at room temperature in the dark. These cells were analyzed with a Muse cell analyzer (Merck KGaA). Annexin V-phycoerythrin (PE)-positive apoptotic and 7-amino-actinomycin D (AAD)-positive necrotic cells were detected by double staining using Annexin V-PE and 7-AAD.

### 2.7. Cell Cycle Analysis

The cell cycle distribution at each phase was measured by staining with propidium iodide (PI). Trypsinized cells were harvested by centrifugation at 500× *g* for 7 min at 4 °C, and then fixed overnight at −20 °C with ice-cold 70% ethanol. Cells (1 × 10^6^) were washed with 1× phosphate-buffered saline (PBS) and incubated with 200 µL of Muse Cell Cycle Reagent (cat. no. MCH100106; Merck KGaA) containing PI and RNase for 30 min at room temperature in the dark. Data from 10,000 cells were analyzed using MACSQuant analyzer and MACSQuantify software version 2.5 (Miltenyi Biotec GmbH).

### 2.8. HK2-Targeting siRNA Transfection

RNA interference of HK2 was performed using a HK2-targeting small interfering RNA (siHK2) duplex from Invitrogen (Oligo ID, HSS179239). Cells (1 × 10^5^) were seeded into 6-well plates and transfected at 40% confluency with siHK2 using Lipofectamine^TM^ RNAiMAX Transfecton Reagent (Invitrogen) according to the manufacturer’s recommendations. Stealth RNAi negative control duplex (siC, Oligo ID, 452001) was used as a negative control. 250 µL. Two hundred and fifty µL of Opti-MEM medium containing siRNAse (25 pmol) and 7.5 µL of Lipofectamine^TM^ RNAiMAX Transfecton Reagent was added to each well. At 24 h after transfection, cells were treated with or without curcumin for another 48 h and then harvested with trypsin for ΔΨ measurement, Annexin V-PE binding assay, cell cycle analysis, and Western blot analysis.

### 2.9. Spheroid Culture and Viability Assay

Spheroid culture was performed in ultra-low attachment 96-well plates, as previously described [24]. Plates seeded with 1 × 10^4^ cells/well were centrifuged at 500× *g* for 10 min to allow cells to cluster in the wells, and then maintained in complete DMEM containing lactic acid for 5 days. Spheroids were treated with curcumin (40 µM) and docetaxel (40 nM) for 48 h. To detect live and dead cells, the green fluorescence of fluorescein diacetate (FDA; Sigma-Aldrich, 5 µg/mL) and red fluorescence for PI (Sigma-Aldrich, 10 µg/mL) were used, respectively. After staining for 5 min, the spheroids were washed with 1× PBS and observed using a Leica EL6000 fluorescence microscope (Leica Microsystems GmbH, Wetzlar, Germany). Spheroid viability was determined with an Enhanced Cell Viability Assay Kit (Young In Frontier Co., Ltd., Geumcheon, Republic of Korea) according to the manufacturer’s instructions. Briefly, 10 µL of Cellvia solution was added to each well, left at room temperature for 1 h, and then mixed for 1 min until the formed formazan crystal dissolved. The amount of formazan formed by living cells was measured spectrophotometrically at 450 nm using a GloMax-Multi microplate multimode reader (Promega Corporation).

### 2.10. Xenograft Assay Using Nude Mice

Five-week-old male mice *(n* = 9, starting size 22–24 g), BklNbt:BALB/c/nu/nu, were acclimated to specific pathogen-free conditions for 1 week, and fed pellets and water ad libitum. They were then randomly divided into two groups (control, 4 mice; treatment, 5 mice) and injected subcutaneously with PC-3AcT cells (2 × 10^6^) suspended in 0.2 mL 1× PBS. Among these, mice in which xenografts were not formed or were too small were excluded, and as a result, 4 mice and 3 mice were used as the control group and the group administered curcumin and docetaxel together, respectively. Curcumin (15 mg/kg) and docetaxel (0.5 mg/kg) were administered intratumorally at three-day intervals. Body weight and tumor growth were observed every 3 days until the 24th day of the experiment. During the experimental period, the mice were observed for clinical signs of distress, including posture, movement, ease of handling, fur condition, arousal, and diarrhea. There were no restrictions on food, water, or exercise during the experimental period, and no animals were subject to humane endpoints or euthanasia due to extreme pain or clinical symptoms. Mice were euthanized by cervical dislocation. The excised tumors were measured for their volume using a Vernier caliper and stored at −80 °C for Western blot analysis. Tumor volume was calculated with the following formula: volume (mm^3^) = length (mm) × width (mm) × width (mm)/2. The animal study was approved by the Institutional Animal Care and Use Committee of Soonchunhyang University on 8 March 2021 (Approval No. SCH20-0060). Animal welfare and experimental procedures were strictly conducted in accordance with the Experimental Animal Welfare Guidelines of the Animal Experiment Center at Soonchunhyang Institute of Medi-Bio Science (Cheonan, Republic of Korea).

### 2.11. Statistical Analysis

All experimental data were analyzed with SPSS version 17.0 (SPSS, Inc., Chicago, IL, USA) and expressed as the mean ± standard deviation of three independent experiments. Comparison between two groups was performed by one-way analysis of variance and Tukey’s post hoc correction. Statistical significance was considered when the *p*-value was less than 0.05.

## 3. Results

### 3.1. Preadaptation of PC Cells to Lactic Acid Makes Them Tolerate Docetaxel Better with an Increase of Glycolytic Flux

**Figure 1:**
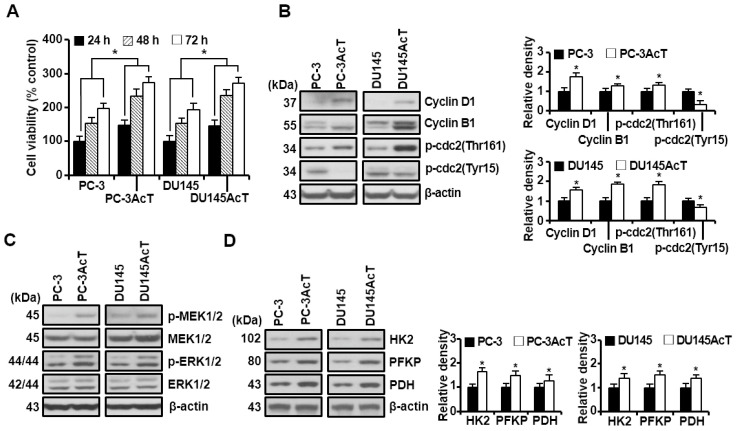

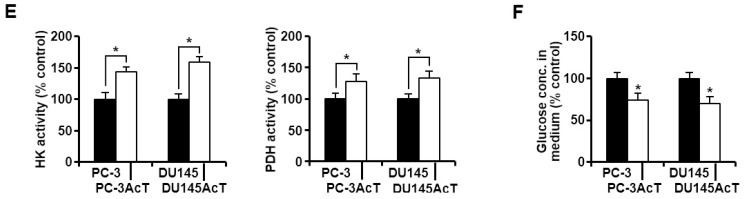
Increased glycolytic flux in PC-3AcT and DU145AcT cells pre-adapted to lactic acid. Cellular responses were examined after culturing cells in DMEM containing 3.8 μM lactic acid for the indicated time (or 48 h, otherwise). (**A**) Percent cell viability. (**B**–**D**) Western blot analysis of cell cycle-regulatory (**B**), MEK/ERK signaling (**C**), and key regulatory enzymes in glycolysis (**D**). (**E**) Activities of hexokinase and pyruvate dehydrogenase. (**F**) Changes in glucose concentration in culture medium. Data are expressed as the mean ± standard deviation of three independent experiments. Statistical significance comparing respective PC-3 or DU145 cells was considered at * *p* < 0.05 using one-way ANOVA and Tukey’s post hoc correction. HK, hexokinase; PFKP, phosphofructokinase platelet; PDH, pyruvate dehydrogenase.

To evaluate the effect of pre-adaptation to lactic acid-containing medium for 15 days, we first measured the growth status and expression of CDKs (p-cdc2Thr161 and p-cdc2Tyr15) and its regulatory subunits (cyclins D1 and B1) of PC-3AcT and DU145AcT cells while culturing them in medium containing 3.8 μM lactic acid. On the first day of culture, some floating cells were observed in the acidic medium, but there was no noticeable difference in the confluency of adherent cells. Compared with their parental PC-3 and DU145 cells, PC-3AcT and DU145AcT cells showed significantly faster growth, with an approximate 1.8-fold and 1.4-fold increase at 48 h and 72 h, respectively (*p* ˂ 0.05, Figure 1A). This was probably as a result of pre-adaptation to acidic medium containing lactic acid, accompanied by upregulation of cyclin D1, cyclin B1, and p-cdc2Thr161, and downregulation of p-cdc2Tyr15 based on Western blot analysis (Figure 1B). The c-Raf/MEK/ERK pathway was also activated in PC-3AcT and DU145AcT cells, as shown by increased levels of p-MEK1/2 and p-ERK1/2 (Figure 1C).

Next, we analyzed proteins or enzymes that are known to play critical roles in aerobic glycolysis and oxidative phosphorylation to investigate the effect of lactic acid on bioenergetics. As shown in Figure 1D, higher levels of HK2, PFKP, and PDH were observed in PC-3AcT and DU145AcT cells than in their parental PC-3 and DU145 cells. After 48 h of culture, the activities of HK and PDH increased (Figure 1E), consistent with results of Western blotting, and the glucose concentration in the culture medium decreased by approximately 74.1% and 69.8% in PC-3AcT and DU145AcT cells compared to PC-3 and DU145 cells, respectively (Figure 1F).

**Figure 2:**
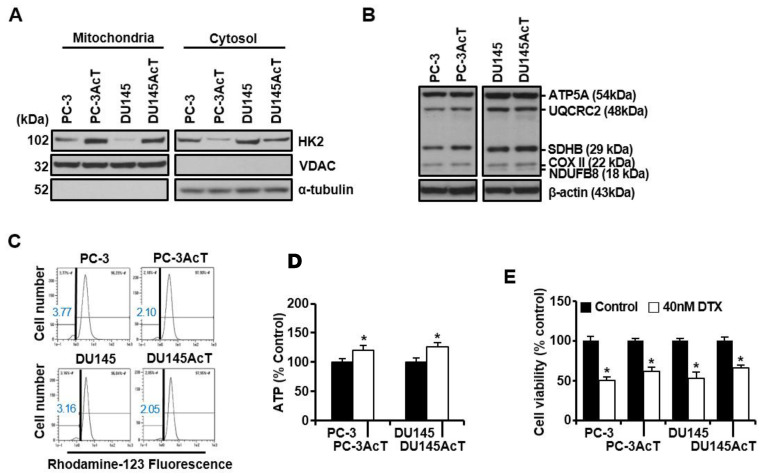

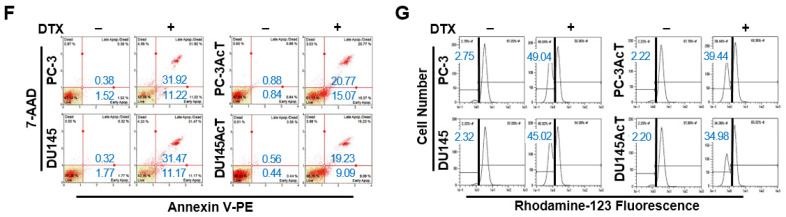
Mitochondrial localization of HK2 and effect of docetaxel treatment on PC-3AcT and DU145AcT cells. Cells were cultured in DMEM containing 3.8 μM lactic acid with or without docetaxel (40 nM) for 48 h. (**A**) Western blot analysis of HK2 in mitochondrial and cytosolic fractions. (**B**) Western blot analysis of complexes I–V in the mitochondrial electron transport chain. (**C**) Measurement of mitochondrial membrane potential after staining cells with rhodamine123. (**D**) Changes in intracellular ATP concentration. (**E**) Percent cell viability for cells treated with or without 40 nM docetaxel. (**F**) Annexin V-PE binding assay for cells treated with or without 40 nM docetaxel. (**G**) Measurements of mitochondrial membrane potential for cells treated with or without 40 nM docetaxel. Data are expressed as the mean ± standard deviation of three independent experiments. Statistical significance comparing respective PC-3 or DU145 cells was considered at * *p* < 0.05 using one-way ANOVA and Tukey’s post hoc correction. HK2, hexokinase 2; VDAC, voltage-dependent anion channel; NDUFB8, NADH-ubiquinone oxidoreductase subunit B8 (complex I); SDHB, succinate dehydrogenase complex iron sulfur subunit B (complex II); UQCRC2, ubiquinone-cytochrome C reductase core protein 2 (complex III); COX II, mitochondrial cytochrome C oxidase subunit II (complex IV); ATP5A, ATP synthase F1 subunit alpha (complex V); DTX, docetaxel.

The proportion of HK2 was increased in the mitochondrial fractions of PC-3AcT and DU145AcT cells compared to PC-3 and DU145 cells (Figure 2A). Base-level analysis of the five complexes in the mitochondrial respiratory chain showed no significant changes except for a slight increase in complex II (SDHB, succinate dehydrogenase subunit B) (Figure 2B). The fractions of cells showing ΔΨ loss, indicative of mitochondrial dysfunction, were slightly reduced in PC-3AcT and DU145AcT cells (Figure 2C). During this process, the ATP contents of these cells were increased significantly, with an approximate 1.2-fold and 1.3-fold increase in PC-3AcT and DU145AcT, respectively (*p* ˂ 0.05, Figure 2D). In the viability assay of PC-3AcT and Du145AcT cells treated with 40 nM docetaxel, both cell lines (approximately 39% and 34% growth inhibition in PC-3AcT and DU145AcT, respectively) showed more resistance to docetaxel than their parental cells (approximately 49% and 47% growth inhibition in PC-3 and DU145, respectively) (*p* ˂ 0.05, Figure 2E). In line with this, the percentage of Annexin V-PE positive cells, indicating an apoptotic fraction, and the percentage of cells with ΔΨ loss, indicating mitochondrial dysfunction, were low in PC-3AcT and DU145AcT cells in response to docetaxel treatment (Figure 2F,G).

### 3.2. HK2-Targeting siRNA or Curcumin Inhibits Growth of Glycolytic and Docetaxel-Resistant PC Cells by Inducing Both Apoptosis and Necroptosis

**Figure 3:**
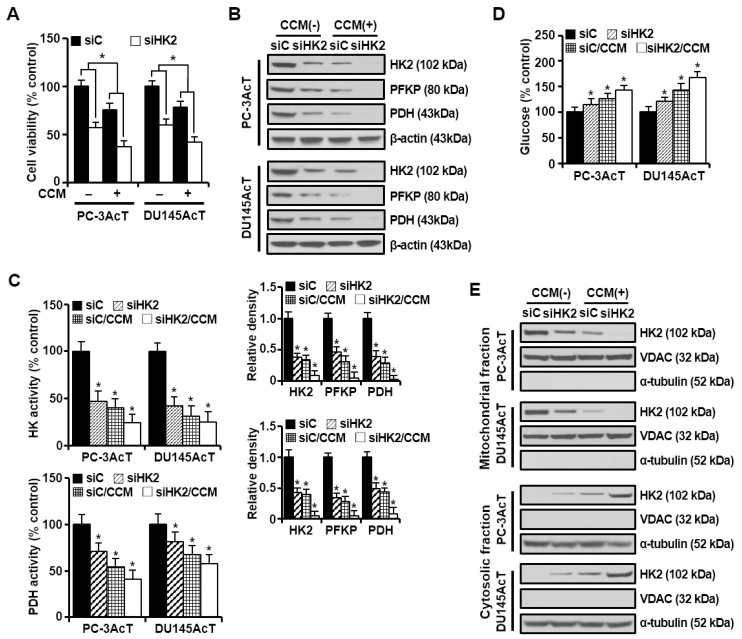
Effect of HK2 knockdown alone or in combination with curcumin on glucose metabolism in PC-3AcT and Du145AcT cells. Cells were transfected with 10 nM HK2-targeting siRNA (siHK2) or stealth RNAi control (siC) for 24 h. They were then treated with or without curcumin (40 μM) in DMEM containing 3.8 μM lactic acid for 48 h. (**A**) Percent cell viability. (**B**) Western blot analysis of key regulatory enzymes in glycolysis. (**C**) Activities of hexokinase and pyruvate dehydrogenase. (**D**) Changes in glucose concentration in culture medium. (**E**) Western blot analysis of HK2 in mitochondrial and cytosolic fractions. The bar graph represents densitometric analysis of Western blot images normalized to β-actin. Data are expressed as the mean ± standard deviation of three independent experiments. Statistical significance comparing the respective siC group was considered at * *p* < 0.05 using one-way ANOVA and Tukey’s post hoc correction. CCM, curcumin; HK, hexokinase; PFKP, phosphofructokinase platelet; PDH, pyruvate dehydrogenase.

In our previous paper, we showed that lactate-acclimated PC-3AcT cells (IC_50_ = 109.57 nM) were more resistant to docetaxel than parental PC-3 cells (IC_50_ = 49.83 nM), whereas the increased sensitivity to curcumin was more evident in PC-3AcT cells (IC_50_ = 42.19 μM) compared to PC-3 cells (IC_50_ = 80.51 μM) [21]. Furthermore, we also found that the preferential cytotoxicity of curcumin against PC-3AcT cells was associated with the inhibition of the glycolytic pathway [22]. To investigate the importance of HK2 as a critical modulator of glycolytic flux in the efficacy of curcumin to preferentially target PC-3AcT and Du145AcT cells, which exhibit a glycolytic and docetaxel-resistant phenotype, we transfected cells with HK2-targeting siRNA, after which cells were treated with vehicle (DMSO) or curcumin for 48 h. HK2 silencing alone induced a growth inhibition of approximately 43% and 40% in PC-3AcT and Du145AcT cells, respectively, compared with the siC group, and the addition of curcumin significantly induced a growth inhibition of approximately 62% and 58%, respectively (*p* ˂ 0.05, Figure 3A). After HK2 was effectively knocked down, the expression level of HK2 decreased to approximately 35% and 37.8% in PC-3AcT and Du145AcT cells, respectively. After 48 h of culture, the expression levels of HK2 and PDH (Figure 3B) also decreased, along with decreased activities of HK and PDH (Figure 3C). Glucose concentration in the culture media remained increased by 14.9% and 21.6% in PC-3AcT and Du145AcT cells with HK2 knockdown, respectively, compared to the siC group (Figure 3D). In line with this, the amount of HK2 in the mitochondrial fraction was also reduced by HK2 silencing (Figure 3E).

**Figure 4:**
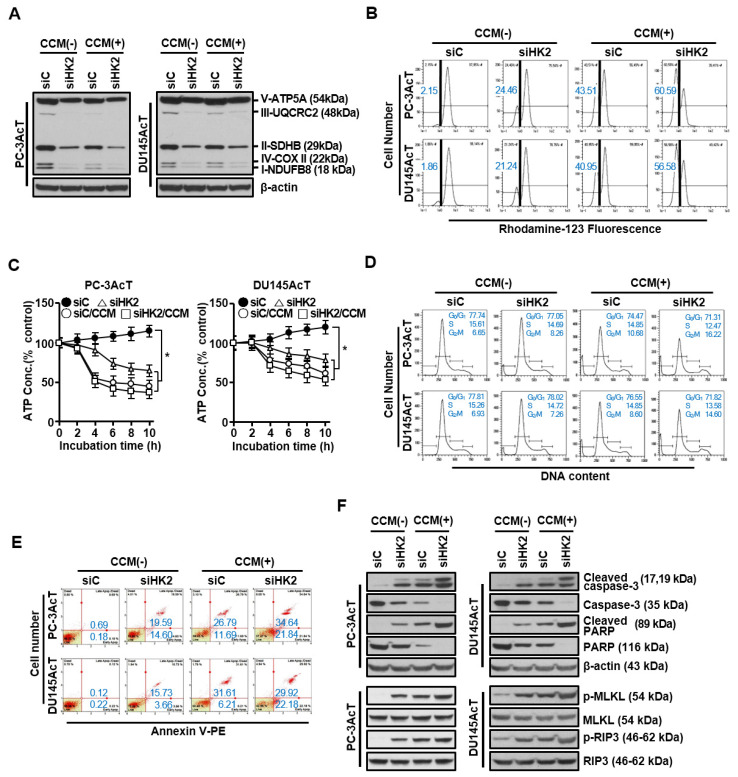
Effects of HK2 knockdown alone or in combination with curcumin on mitochondrial function and programmed cell death in PC-3AcT and Du145AcT cells. Cells were transfected with 10 nM HK2-targeting siRNA (siHK2) or stealth RNAi control (siC) for 24 h. They were then treated with or without curcumin (40 μM) in DMEM containing 3.8 μM lactic acid for 48 h. (**A**) Western blot analysis of complexes I–V in mitochondrial electron transport chain. (**B**) Measurements of mitochondrial membrane potential after staining cells with rhodamine123. (**C**) Changes in intracellular ATP concentration. (**D**) Cell cycle analysis. (**E**) Annexin V-PE binding assay. (**F**) Western blot analysis of apoptosis- and necroptosis-related proteins. Data are expressed as the mean ± standard deviation of three independent experiments. Statistical significance comparing the respective siC group was considered at * *p* < 0.05 using one-way ANOVA and Tukey’s post hoc correction. CCM, curcumin; NDUFB8, NADH-ubiquinone oxidoreductase subunit B8 (complex I); SDHB, succinate dehydrogenase complex iron sulfur subunit B (complex II); UQCRC2, ubiquinone-cytochrome C reductase core protein 2 (complex III); COX II, mitochondrial cytochrome C oxidase subunit II (complex IV); ATP5A, ATP synthase F1 subunit alpha (complex V).

HK2 silencing alone or in combination with curcumin treatment downregulated the expression of complexes I–V in the respiratory chain (Figure 4A) but increased the proportion of cells with ΔΨ loss, indicative of mitochondrial dysfunction (Figure 4B). During this process, the ATP content of these cells was significantly reduced in the following order: HK2 silencing alone ˂ curcumin ˂ HK2 silencing/curcumin (*p* ˂ 0.05, Figure 4C). Flow cytometric analysis of cells with HK2 knocked down using HK2-targeting siRNA showed an increase in the sub-G_0_/G_1_ peak, indicative of apoptosis (Figure 4D), accompanied by an increase in the proportion of apoptotic cells as determined by Annexin V-PE binding assay (Figure 4E). Consistently, HK2 silencing induced enhanced cleavage of procaspase-3 and its substrate PARP, and increased the phosphorylation levels of MLKL and RIP3 (Figure 4F) compared with the siC group. The effects of HK2 silencing alone were further enhanced by the addition of curcumin.

### 3.3. Combination Treatment with Curcumin and Docetaxel Inhibits Glycolysis but Increases Both Apoptosis and Necroptosis in 3D Spheroid Cultures and PC-3AcT Xenografts

**Figure 5:**
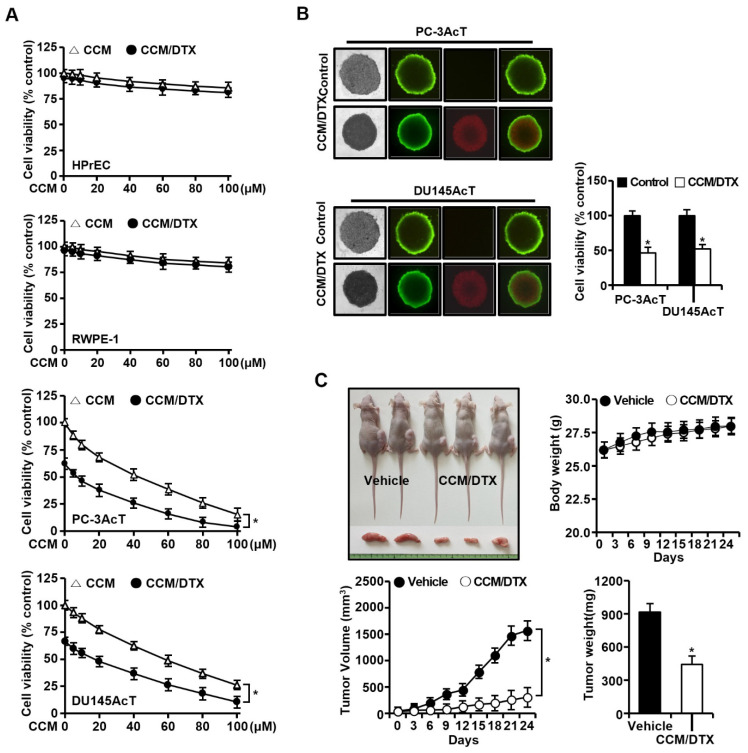
Growth-inhibiting effect of co-treatment with curcumin and docetaxel. (**A**) Cell viability in 2D monolayer culture. Cells were cultured in DMEM containing 3.8 μM lactic acid with or without curcumin (40 μM) and docetaxel (40 nM) for 48 h. (**B**) Vitality staining of spheroids: from left to right: (i) phase-contrast image, (ii) fluorescent images of fluorescein diacetate(+) living cells in green, (iii) propidium iodide(+) dead cells in red, and (iv) merged; and spheroid cell viability. Spheroids were then treated with or without curcumin (40 µM) and docetaxel (40 nM) for 48 h in DMEM containing 3.8 μM lactic acid. (**C**) Representative mice, body weight, tumor volume, and tumor weight in PC-3-xenografted nude mice model. Mice (0.3–0.4 cm wide and 0.3–0.4 cm long) were injected intratumorally with vehicle or curcumin (15 mg/kg) and docetaxel (0.5 mg/kg) three times per week for 24 days. Data are expressed as the mean ± standard deviation of three independent experiments. Statistical significance comparing the respective control group was considered at * *p* < 0.05 using one-way ANOVA and Tukey’s post hoc correction. CCM/DTX, co-treatment with curcumin and docetaxel.

To confirm the consistency of the results in 2D monolayer cultures and to assess whether co-treatment with docetaxel affected the efficacy of curcumin, we investigated the effect of combined treatment with docetaxel and curcumin in 3D spheroid cultures and a nude mouse xenograft model. In 2D monolayer cultures, curcumin treatment at concentrations minimally toxic to normal prostate epithelial cells (HPrEC and RWPE-1) reduced cell viability in a concentration-dependent manner, and the effect was further increased by combined treatment with docetaxel (Figure 5A). Curcumin (40 µM) alone or in combination with docetaxel (40 nM) exhibited growth inhibition of approximately 48% and 74% in PC-3AcT, and 38% and 63% in DU145AcT, respectively. The combination index calculated by treating curcumin (40 uM) and docetaxel (40 µM) was 0.711 in PC-3AcT and 0.715 DU145AcT, suggesting that combination treatment synergistically inhibited cell growth. Consistent with the 2D monolayer culture results, combined treatment with curcumin and docetaxel increased the red fluorescence of PI inside the spheroid, representing the necrotic core, but decreased the green fluorescence of FDA, representing surrounding viable cells, and inhibited spheroid growth (Figure 5B). Cell viability of 3D spheroids also decreased to 46.5% and 51.9% in PC-3AcT and Du145AcT cells, respectively, compared to the control (Figure 5B). In a xenograft mouse model of PC-3AcT cells, one week after subcutaneous inoculation of cancer cells (2 × 10^6^) into male nude mice, curcumin (15 mg/kg) and docetaxel (0.5 mg/kg) were administered intratumorally every three days for a total of 24 days. PC-3AcT tumor volumes grew progressively in mice. However, the combination treatment with the agents significantly reduced tumor volume and weight during the experiments without apparent toxicity, as determined by body weight and appearance (posture, movement, and fur condition, etc.) of mice, indicating that the tumor-bearing mice tolerated treatment fairly well (Figure 5C).

**Figure 6:**
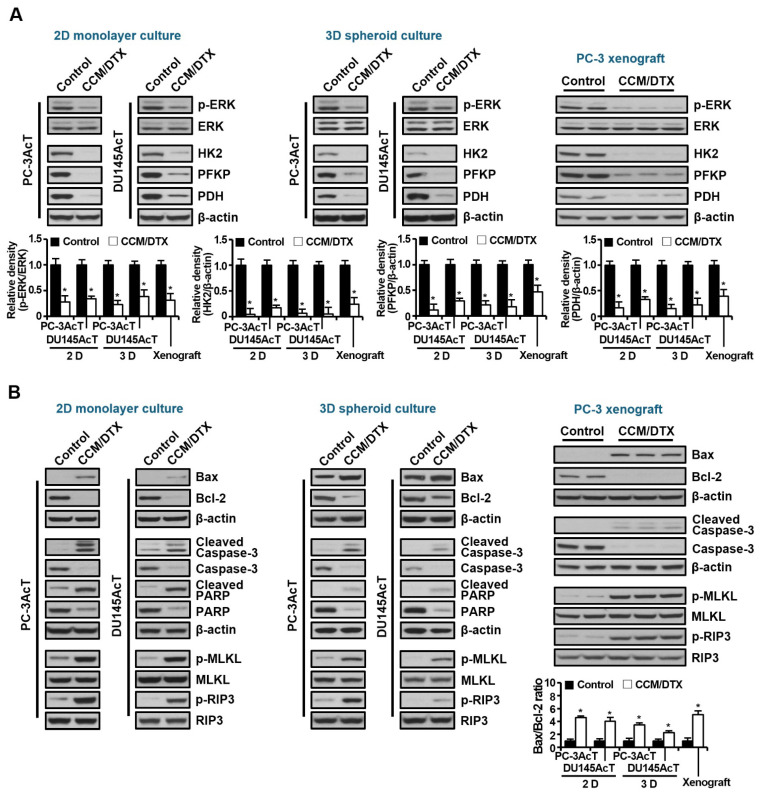
Effects of co-treatment with curcumin and docetaxel on expression of glycolysis-, apoptosis-, and necroptosis-related key proteins in 2D monolayer, 3D spheroid cultures, and nude mice xenograft models. Proteins were extracted from cells, spheroids, and tumors described in Figure 5, separated on 4–12% NuPAGE gels, and subjected to Western blot analysis. (**A**) Expression levels of key regulatory enzymes of glycolysis. (**B**) Expression levels of proteins related to apoptosis and necroptosis. The bar graph represents densitometric analysis of Western blot images normalized to β-actin. Data are expressed as the mean ± standard deviation of three independent experiments. Statistical significance comparing the respective control group was considered at * *p* < 0.05 using one-way ANOVA and Tukey’s post hoc correction. CCM/DTX, co-treatment with curcumin and docetaxel.

Next, we compared the effects of curcumin and docetaxel on the expression of key enzymes in glycolysis and programmed cell death. As shown in Figure 6A, downregulation of the expression levels of HK2, PFKP, and PDH, as well as the phosphorylation level of ERK1/2 was observed in both 2D and 3D cultures or tumor tissues in nude mouse xenograft models treated with the combination of two agents (Figure 6A). Additionally, along with an increase in the Bax/Bcl-2 ratio, cleaved caspase-3 and cleaved PARP, markers for apoptosis, and p-MLKL and p-RIP3, markers for necroptosis, were also upregulated by the combination treatment (Figure 6B). The above results indicate that our in vivo data reflect the in vitro results, and that the addition of docetaxel did not affect the antiglycolytic and cell killing effects of curcumin.

## 4. Discussion

We have previously observed preferential cytotoxicity of curcumin against lactate-acclimated PC-3AcT cells, which showed a higher tolerance to docetaxel than parental PC-3 cells [20]. In this pre-adaption process, lactic acid might promote resistance to docetaxel, whereas curcumin was shown to induce ROS generation, DNA damage, and subsequent mitochondrial dysfunction, ultimately leading to apoptosis and necroptosis of PC-3AcT cells. In the present study, despite enhanced glycolysis in PC-3AcT and DU145AcT compared to parental PC-3 and DU145 cells, the basal levels of the five complexes of the mitochondrial respiratory chain and mitochondrial membrane potential were not significantly increased. This suggests that lactate-adapted PC cells have more metabolic dependence on glycolysis for ATP synthesis than on mitochondrial respiration. At basal levels, PC-3AcT and DU145AcT cells exhibited the following characteristics compared to PC-3 and Du145 cells: (i) increased cell growth with up-regulation of cyclin D1, cyclin B1, and p-cdc2^Thr161^ but down-regulation of p-cdc2^Tyr15^, (ii) activation of the c-Raf/MEK/ERK pathway, (iii) increased glucose consumption along with increased levels and activities of key regulatory enzymes in glucose metabolism, including HK2, and (iv) increased tolerance to docetaxel.

Cell cycle progression is mediated by the sequential activation of Cdks in association with their regulatory cyclin subunits, in which phosphorylation or dephosphorylation by Cdk inhibitors is a major mechanism regulating the activity of Cdks [25]. Upregulation of cell cycle regulatory proteins cyclin D1 and cyclin B1 is vital in cell cycle progression in the G_0_/G_1_ phase and G_2_/M transition, respectively. Activation of cdk1 (previously called cdc2) through phosphorylation of Thr161 and dephosphorylation of Thr14/Tyr15 also promotes subsequent mitotic entry in G_2_ phase [26,27], ensuring faster proliferation of PC-3AcT and DU145AcT cells. The ERK1/2 pathway is activated by sequential reactions of upstream factors p-Raf and p-MEK1/2. In addition to its well-known role in regulating cell proliferation and survival, the involvement of ERK1/2 signaling in promoting the Warburg-like metabolism has also been reported by some researchers [28,29]. The MEK/ERK1/2 pathway not only increases the expression of glucose Transporter 1, lactate dehydrogenase A, and several enzymes in the glycolysis pathway through the role of the transcription factor c-Myc, but also cooperates with hypoxia-inducible factor-1α to induce HK2 [28]. Cui et al. have reported that HK2 upregulates cyclin A1 and downregulates p27 by activating ERK1/2 through the Raf/MEK signaling pathway, further promoting proliferation and tumor formation of cervical cancer cells [29]. Our previous study has shown that ERK1/2 inhibition by PD98059 (a synthetic inhibitor of MEK1/MEK2) or curcumin could reduce glucose consumption and downregulate the expression of key regulatory enzymes of glucose metabolism, including HK2, leading to apoptosis and necroptosis in PC-3AcT and Du145AcT cells [22]. These results demonstrated by us and others highlight the importance of the Raf/MEK/ERK pathways in promoting the Warburg effect.

In this study, docetaxel resistance in PC-3AcT and DU145AcT cells was manifested by improved cell viability, reduced apoptosis, and mitochondrial dysfunction compared to their parental cell lines PC-3 and Du145. Although multiple mechanisms are involved in the resistance of cancer cells to chemotherapy, our study suggests that increased aerobic glycolysis is associated with docetaxel resistance in lactate-acclimated PC cells. Aberrant expression of key regulators of glycolysis, including HK2, pyruvate kinase, PDH, and LDH, contributes to tumorigenesis and chemoresistance [30]. Additionally, activation of the pentose phosphate pathway through increased glycolytic flux provides reducing equivalents for protection against ROS [3], which may prevent or reduce oxidative damage caused by docetaxel in PC-3AcT and DU145AcT cells. Therefore, increased metabolic dependence of PC-3AcT and DU145AcT cells on glycolysis might prevent or reduce docetaxel-induced mitochondrial damage and ROS levels.

Overexpression of HK2 during tumor growth is associated with poor patient prognosis, disease progression, metastasis, and treatment resistance in various malignancies [31,32]. In particular, the localization and attachment of HK2 to the outer mitochondrial membrane allow tumor cells to cope with many stressful conditions by intersecting both metabolic and anti-apoptotic pathways [32]. Binding of HK to the VDAC can facilitate the release of mitochondrial-generated ATP, making it more readily available for glucose phosphorylation. It can also stabilize the mitochondrial permeability transition pore (MPTP), providing cells with protection against apoptotic stimuli [31,32]. Conversely, HK2 deficiency by knockdown or knockout of HK2 impaired tumor growth or increased treatment sensitivity. HK2 knockout can inhibit tumorigenesis in mouse models of KRAS-driven lung cancer [33], ErbB2-driven breast cancer [34], and PTEN-deficient PC [35]. In a mouse model of hepatocarcinoma, deletion or silencing of hepatic HK2 has resulted in reduced tumorigenesis and increased sensitivity to metformin or sorafenib [36]. Consistent with these reports, we found that HK2-targeting siRNAs clearly showed a significantly enhanced cell killing effect against both PC-3AcT and DU145AcT cells. A reduction in cellular ATP content due to the inhibition of both glycolysis and mitochondrial function appeared to be the main cause that led to induction of apoptosis and necroptosis. In this process, the effect of curcumin and HK2 silencing appear to be functionally additive, with curcumin’s anti-glycolytic role further strengthening the effect of HK2 silencing on PC-3AcT and DU145AcT cells. These data demonstrate that HK2 has essential functions in regulating the survival and growth of PC-3AcT and DU145AcT cells, suggesting the potential significance of HK2 targeting by curcumin as a promising strategy to improve sensitivity to docetaxel.

The preferential cytotoxic effect of curcumin on PC-3AcT cells, which showed an increased tolerance to docetaxel in our previous study [21], and the better killing effect of combined treatment with docetaxel and curcumin than either docetaxel or curcumin observed in this study suggest the potential of curcumin as an adjunctive treatment to docetaxel. The chemo-potentiating effect of curcumin has been shown to be effective without increasing toxicity when used in combination with other established chemotherapeutic agents, such as docetaxel for the treatment of metastatic castration-resistant PC [37], cisplatin in bladder cancer lines [38], doxorubicin in Hodgkin’s lymphoma-derived cell line L540 [39], 5-fluorouracil in colorectal cancer [40], and metformin in hepatocellular carcinoma and PC [41,42]. Prior to a full-scale preclinical evaluation of the combination therapy of curcumin and the anticancer drug docetaxel in animal models, we had the premise that the antiglycolytic efficacy of curcumin should not be affected by the addition of docetaxel. In this study, curcumin combined with docetaxel at concentrations that exhibited minimal cytotoxicity to normal prostate epithelial cells induced a significant cell death of PC-3AcT and DU145AcT, as evidenced by anti-glycolytic, apoptotic, and necroptotic effects. The response of cells to combined treatment observed in conventional monolayer cultures was similar in 3D spheroid cultures and nude mouse xenograft models. In particular, the antiglycolytic effect of curcumin in 3D spheroid and xenografts was not affected in the presence of docetaxel, as molecular analysis showed only minor or non-significant differences compared to the in vitro effect in monolayer culture. Three-dimensional (3D) spheroid cultures, which better mimic the in vivo microenvironment, generally tend to be more resistant to anticancer treatment than 2D monolayer cultures. Several factors that lead to different responses in 3D cultures relative to the 2D cultures include the accumulation of more quiescent cells in the center of the spheroid, resulting in increased resistance to chemotherapy [43], enhancement of signaling pathways that participate in drug resistance due to cell–cell or cell–matrix interactions [44], and poor accessibility of drugs inside the spheroid [45,46]. In addition, cells in the hypoxic core of spheroids (which are also seen in solid tumors) are more resistant to therapy and more dependent on anaerobic glycolysis, so oxygen availability may influence cell viability and expression of glycolytic enzymes in response to treatment. In this study, the anti-glycolytic and cytotoxic effects of curcumin and docetaxel in 3D spheroid cultures and xenograft models closely resembled their in vitro effects in monolayer cultures, with only minor or non-significant differences in molecular analysis. Our in vitro experimental conditions, which mimic the tumor microenvironment such as the slightly acidic nature of the lactic acid-containing medium (pH 6.8–7.0), might have minimized these differences. This result also supports a previous report showing that the microenvironment plays a significant role in cellular responses to drugs [47]. Therefore, studies using lactate-acclimated cell lines might be a useful strategy for understanding the role of lactic acid in the tumor microenvironment and developing drugs targeting it. To maintain consistency of drug responses between in vitro and in vivo experiments, it is necessary to introduce experimental conditions that mimic the tumor microenvironment, including a hypoxic condition. Together, we plan to conduct more intensive animal model experiments that take into account in vivo drug efficacy, dose optimization, administration route, toxicity testing, and safety testing, based on our validated in vitro experimental results.

## 5. Conclusions

Our results provide evidence that lactic acid may play a role in protecting cells against docetaxel through activation of glycolysis and the MEK/ERK pathways. Additionally, by mechanistically elucidating the role of curcumin in inhibiting these pathways, our results support the findings of a previous study revealing preferential cytotoxicity of curcumin against PC-3AcT cells showing increased tolerance to docetaxel. The chemo-potentiating effect of curcumin on PC cells displaying a glycolytic phenotype may help it exert profound cell killing effects by activating both apoptosis and necroptosis.

## Data Availability

All data produced or assessed during this study are included in this article.
